# A New theraphosid Spider Toxin Causes Early Insect Cell Death by Necrosis When Expressed *In Vitro* during Recombinant Baculovirus Infection

**DOI:** 10.1371/journal.pone.0084404

**Published:** 2013-12-13

**Authors:** Daniel Mendes Pereira Ardisson-Araújo, Fabrício Da Silva Morgado, Elisabeth Ferroni Schwartz, Gerardo Corzo, Bergmann Morais Ribeiro

**Affiliations:** 1 Departmento de Biologia Celular, Universidade de Brasília, Brasília, Brasília, DF, Brazil; 2 Departmento de Ciêncais Fisiológicas, Universidade de Brasília, Brasília, DF, Brazil; 3 Departamento de Medicina Molecular y Bioprocesos, Universidad Nacional Autónoma de México, Cuernavaca, Morelos, México; Instituto de Biotecnología, Universidad Nacional Autónoma de México, Mexico

## Abstract

Baculoviruses are the most studied insect viruses in the world and are used for biological control of agricultural and forest insect pests. They are also used as versatile vectors for expression of heterologous proteins. One of the major problems of their use as biopesticides is their slow speed to kill insects. Thus, to address this shortcoming, insect-specific neurotoxins from arachnids have been introduced into the baculovirus genome solely aiming to improve its virulence. In this work, an insecticide-like toxin gene was obtained from a cDNA derived from the venom glands of the theraphosid spider *Brachypelma albiceps*. The mature form of the peptide toxin (called Ba3) has a high content of basic amino acid residues, potential for three possible disulfide bonds, and a predicted three-stranded β-sheetDifferent constructions of the gene were engineered for recombinant baculovirus *Autographa californica multiple nuclepolyhedrovirus* (AcMNPV) expression. Five different forms of Ba3 were assessed; (1) the full-length sequence, (2) the pro-peptide and mature region, (3) only the mature region, and the mature region fused to an (4) insect or a (5) virus-derived signal peptide were inserted separately into the genome of the baculovirus. All the recombinant viruses induced cell death by necrosis earlier in infection relative to a control virus lacking the toxin gene. However, the recombinant virus containing the mature portion of the toxin gene induced a faster cell death than the other recombinants. We found that the toxin construct with the signal peptide and/or pro-peptide regions delayed the necrosis phenotype. When infected cells were subjected to ultrastructural analysis, the cells showed loss of plasma membrane integrity and structural changes in mitochondria before death. Our results suggest this use of baculovirus is a potential tool to help understand or to identify the effect of insect-specific toxic peptides when produced during infection of insect cells.

## Introduction

Insects are a major cause of reduction in crop yields and currently chemical insecticides are still the dominant method for controlling pest populations [1]. However, due to the negative environmental impact of chemical insecticides and the appearance of resistant insects, the search for alternative methods of controlling insect pests has increased. Biological control methods such as insecticidal toxins, present in many venomous organisms [2,3] have been shown to be a reasonable option for replacing chemical agents [4]. Arachnids harbor one of the most attractive arsenal of peptides with high toxicity and specificity for insects [5–8], providing a potential source for development of biological pesticides [9,10]. For spiders, in particular, which are natural insect predators, proteomic analyses have revealed that venoms at some species may contain more than 1,000 unique peptides [11]. Spider venom peptides are commonly rich in disulfide bonds and have been found to be one of the major contributors to the insecticidal activity [12]. However, the low viability of venom and the difficulty of peptide purification have hampered application of insecticidal peptides in insect pest control [12].

Heterologous expression systems are an alternative choice for production of bioactive peptides rather than organism extraction. However, the choice of the expression system needs to ensure a correct expression of the desired peptide [13–15]. An most attractive way of ensuring an appropriate expression system is to use methods in which the heterologously expressing cell is related to the gene of interest of the organism-source [16] (i.e. insect). Therefore, the use of baculovirus and insect cells, a largely and well established eukaryotic expression system, allows an arthropod-related cell environment for the expression of arachnid peptides [17–19].

Baculoviruses are insect viruses that have been widely used as expression vectors for heterologous proteins in insect cells since the early 80’s [20]. Since then, thousands of recombinant proteins have been expressed in insect cells for numerous different applications using different strategies [21,22]. Furthermore, baculovirus could be also used as biological control agents. Interestingly, during a complete baculovirus infection cycle, two different forms of virions are produced: an occlusion-derived virus (ODV) and a budded virus (BV). ODVs are encased in occlusion bodies (OB) called polyhedra which are dispersed in the environment naturally upon insect death caused by the virus. Insects feed on polyhedra-contaminated leaves and are infected *per os* by OB-released ODVs, which establish a primary infection in the insect midgut cells [23]. After the infection of midgut cells, the BV phenotype is produced early on infection and are responsible for the secondary infection of all other host tissues [24]. Additionally, engineered baculoviruses expressing entomotoxic peptides have been used mainly for biological control study purposes [25–31], and in the last few years, active spider proteins produced in baculovirus/insect cell system have been functionally characterized [18,19].

Insecticidal toxins target a wide range of targets in insect cells and therefore, generate different cell responses [12]. The study of insect cell responses to different toxins expressed during infection by recombinant baculoviruses could help us understand the molecular mechanism of action of these toxins. Thus, in this work, we constructed recombinant baculoviruses containing different variants of a newly identified spider toxin gene isolated from the Mexican theraphosid *Brachypelma albicebs* Pocock, 1903. The venom from this spider has high insecticidal activity and its composition is quite understood [32,33]. Thus, the recombinant baculoviruses were used to infect insect cells and to evaluate the effects of the toxin expressed through structural and ultrastrutural analyses of host cells during virus infection.

## Material and Methods

### Viruses and insect cell lines

*Trichoplusia ni* (cabbage looper) BTI-Tn5B1-4 (Tn5B) cells [34] and *Spodoptera frugiperda* (fall armyworm) IPLB-Sf21-AE (Sf21) cells [35] were maintained at 28 °C in TC-100 medium (HIMEDIA), supplemented with 10% fetal bovine serum (Invitrogen, Carlsbad, CA, USA), and with an antibiotic-antimycotic mixture (Gibco, Carlsbad, CA, USA). Recombinant viruses derived from AcMNPV were propagated in insect cell cultures and their titers determined according to O’Reilly [36].

### cDNA library construction and Ba3 gene cloning

A cDNA library was constructed from mRNA extracted from a pair of venomous glands from one specimen of *B. albiceps*. Briefly, the mRNA was extracted from the two venom glands, and reverse-transcribed and amplified employing theTotal RNA Isolation System kit (Promega, Madison, WI, USA)[37]. With this material, a full-length cDNA plasmid library was prepared using the SMART cDNA Library Construction Kit (CLONTECH Lab., Palo Alto, CA). The amplified cDNAs were ligated with a pCR TOPO II TA cloning vector (Invitrogen, San Diego, CA, USA) and transformed into TOP 10 competent cells (Invitrogen, San Diego, CA, USA) followed by overnight culturing on 50 µg/mL ampicillin-containing LB plates at 37°C. The resulting colonies were picked randomly and the insert cDNAs in the individual colonies were directly amplified by PCR using SP6 and T7 primer sets. The PCR products were resolved by agarose gel electrophoresis to determine the size of each product. Based on the information obtained from direct peptide sequencing of Ba1 [33], a specific oligonucleotide (Table S1) was designed and used for the PCR reaction using the cDNA library as a template. The PCR reaction was performed using 1x supplied reaction buffer, 200 µM dNTPs, 0.25 µM forward primer (FA) 0.25 µM; reverse primer (CDS3) and 2 units of Vent DNA polymerase in a Perkin Elmer 9600 instrument (Waltham MA, USA). The reaction was incubated at 94°C for 5 min, and at 50°C for 7 min before Taq DNA polymerase was added. The mixture was then incubated at 94°C for 3 min for one cycle. After the initial cycle, the mixture was incubated at 94°C for 30 s, 60°C for 1 min and 72°C for 1 min per 30 cycles, followed by a final 7 min step at 72°C. PCR products were purified using high pure PCR product purification Kit (Roche, Basel,Switzerland) following the manufacturer instructions, and then ligated into a TOPO 2.1 (Invitrogen, Carlsbad, CA, USA) digested plasmid. The ligation reaction was used to transform competent *E. coli* XL1-blue competent cells (Stratagene, Santa Clara, CA, USA). Positive clones were sequenced from both ends using the Thermo sequenase radiolabeled terminator cycle sequencing kit (Amersham, Piscataway, NJ,USA). The Ba3 gene was obtained from among these clones. GenBank accession number is KF638632.

### Computational analysis

Toxin sequence analysis was carried out using the SignalP 4.1 Server (http://www.cbs.dtu.dk/services/SignalP/) for signal peptide prediction. The Swiss-Model Server (http://swissmodel.expasy.org/) was used for protein structure prediction using NMR-resolved theraphotoxin Ba2, previously found in the crude venom of the same spider (pdb: 2kgh) as base model. The cysteine disulfide bond prediction was performed with the Predict Protein server (www.predictprotein.org). Peptide alignment was performed using MAFFT method [38].

### Toxin gene amplification and construction of shuttle vectors

Five PCRs using different sets of primers (Table S1) and the cDNA library as template, were carried out to generate different variants of the *B. albiceps* toxin gene (*ba3*): F1 and R to amplify the full-length toxin gene (pre-propeptide, *sp-pp-ba3*); F2 and R to amplify the propeptide gene (*pp-ba3*); F3 and R to amplify the mature toxin gene (*ba3*); F4 and R to generate the mature toxin gene fused to a baculovirus-derived signal peptide (*spe-ba3*); and F5 and R to generate the mature toxin gene fused to an insect-derived signal peptide (*spb-ba3*). The forward primers F1 to F5 added to the amplified gene sequences for *Bam*HI restriction enzyme (underlined in the primer sequence in Table S1) and start codon when needed (in italics). The F4 and F5 oligonucleotides added signal peptide sequences (showed in lower case) derived from the *Anticarsia gemmatalis multiple nucleopolyhedrovirus* (AgMNPV) *ecdysteroid UDP–glucosyltransferase* (*egt*) gene [39] and *Bombyx mori bombyxin* gene [40], respectively. The reverse primer R added a *Hin*dIII site (underlined). The five amplification reactions contained 50 ng of the DNA-template, 300 µM of dNTP mix (Fermentas, Pittsburgh, PA, USA), 0.4 µM of each set of the related primer pairs, 1 U of Platinum® Taq DNA Polymerase (Invitrogen, Carlsbad, CA, USA), and 1x of the supplied reaction buffer. The reactions were subjected to the following program: 94 °C/5 min, 35 cycles of 94 °C/ 30 s, 60 °C/20 s and 72 °C/20 s with a final extension of 7 min at 72°C using Swift™ Maxi Thermal Cycler (ESCO Technologies). The fragments obtained were analyzed by electrophoresis in 1.5 % agarose gels [41], purified using the GFX PCR DNA and Gel Band Purification kit (GE Healthcare, Piscataway, NJ,USA), cloned into the pGem®-T easy vector (Promega, Madison, WI, USA), and sequenced (Macrogen, Seoul, Korea).

All confirmed plasmids were digested with *Bam*HI and *Hin*dIII (Promega, Madison, WI, USA) according to manufacturer’s instructions. The reactions were analyzed by electrophoresis in 1.5 % agarose gels [41] and the released fragments containing the different variants of the toxin were purified from the gel as described above, and cloned into the commercial transfer vector pFastBac1® (pFB1 – Invitrogen, Carlsbad, CA, USA) previously digested with the same restriction enzymes.

### Transfer vector modification for bacmid-based occluded virus construction

For the construction of a occlusion positive (occ+) recombinant virus harboring different variants of the toxin gene, the commercial plasmid pFB1 (Invitrogen, Carlsbad, CA, USA) was digested with *Acc*I restriction enzyme (Promega, Madison, WI, USA) to release the *polyhedrin* promoter (Ppolh). The restriction digestion was analyzed by electrophoresis in a 0.8% agarose gel and the fragment without the Ppolh was purified as described above. This fragment was then blunted using Klenow enzyme (Promega, Madison, WI, USA), ligated using T4-DNA ligase (Promega, Madison, WI, USA), and transformed into *Escherichia coli* DH5-α competent cells (Invitrogen, Carlsbad, CA, USA). The pFB1/*Acc*I plasmid generated was confirmed by digestion and was used to clone a DNA fragment from the transfer vector pSyn XIV VI+ X3 [42]. This fragment was called PSX because it contains the AcMNPV *polyhedrin* (**P**) gene under the transcriptional control of its own promoter and two promoters, p**S**yn (**S**) and p**X**IV (**X**) *in tandem* and opposite orientation for heterologous gene expression. The PSX fragment was obtained by PCR using 10 ng of the DNA-template, 300 µM of dNPT mix (Fermentas, Pittsburgh, PA, USA), 0.4 µM of each pSyn-F and pSyn-R primer (Table S1), and 1 U of LongAmpTaq DNA polymerase (New England Biolabs, Ipswich, MA, USA). The reaction was subjected to the following program: 94 °C/5 min, 30 cycles of 94 °C/ 30 s, 55 °C/20 s and 65 °C/50 s, with a final extension for 10 min at 65° C using Swift™ Maxi Thermal Cycler (ESCO Technologies). After amplification, the PSX fragment was digested with *Not*I and *Spe*I (Promega, Madison, WI, USA) and cloned into the pFB1/*Acc*I previous digested with the same restriction enzymes in order to generate the pFastBac1/*AccI*-PSX plasmid. All procedures were carried out according to manufacturer’s instructions. Three different variants of the toxin gene (*spb-ba3*, *spe-ba3*, and *ba3*) were cloned into pFastBac1/*AccI*-PSX *Not*I-digested (Promega, Madison, WI, USA) and dephosphorylated with Shrimp Alkaline Phosphatase enzyme (SAP – Promega, Madison, WI, USA). The heterologous insertion was confirmed by restriction enzyme digestion and sequencing (Macrogen, Seoul, Korea).

### Recombinant virus construction

pFB1 and pFB1/*AccI*-PSX harboring the toxin variants were transformed into DH10-Bac cells (Invitrogen, Carlsbad, CA, USA) by electroporation [41] and recombinant bacmids were selected and confirmed by PCR following the manufacturer’s instructions (Bac-to-Bac®, Baculovirus expression systems, Invitrogen, Carlsbad, CA, USA). DNAs from the bacmids were purified and the presence of the recombinant gene was checked by PCR using specific oligonucleotides as described by the manufacturer (Invitrogen, Carlsbad, CA, USA). One microgram of each recombinant bacmid was transfected into Tn5B cells (10^6^) using liposomes (Cellfectin® - Invitrogen, Carlsbad, CA, USA). Since naked baculovirus DNA is infectious, the supernatant from seven days post-transfection of Tn5B cells containing the recombinant viruses were collected, amplified in the same insect cells and tittered as described in O’Reilly et al. [36].

### Polyhedra production in insects

*S. frugiperda* larvae in early fifth-instar were provided by EMBRAPA/CENARGEN – Genetic Resources and Biotechnology (Brasília, Brazil). During the experiments, the insects were maintained individually in 50 mL transparent plastic vials at 25 °C and fed on artificial diet [43]. The insect infection was carried out by injection of 10 µL of TC-100 medium containing 10^6^ recombinant viruses. For polyhedra production, caterpillars were also infected by injection with occlusion positive recombinant viruses as described above. The insect cadavers were processed according to O’Reilly et al [36] and the polyhedra were counted in a hemocytometer.

### Light and transmission electron microscopy

For light microscopy, monolayers of Sf21 (5 x 10^6^) cells were infected at a multiplicity of infection (moi) of 10. The infected cells were observed and photographed at different hours post-infection in an Axiovert 100 inverted light microscope (Zeiss). For transmission electron microscopy (TEM), Sf21 cells (5 x 10^6^) were infected as above (moi of 10) and at 36 h p.i. were fixed for 2 h (2% glutaraldehyde, 2% paraformaldehyde in 0.1 M sodium cacodylate buffer pH 7.4 with 5% sucrose), post-fixed (1% osmium tetroxide, 0.8% potassium ferricyanide in the same buffer as above), contrasted with 0.5% uranyl acetate, dehydrated in acetone, and embedded in Spurr’s resin. The ultrathin sections were contrasted with uranyl acetate/lead citrate and observed in a TEM Jeol 1011 at 80 kV.

### Cytotoxicity assays

Sf21 insect cells were seeded in 96-well plates (4 x10^4^ cells/well) and infected (moi of 10) with different viruses. At different hours post-infection (0, 24, 48, 72, 96, and 120 h p.i..), viral-induced cytotoxicity was analyzed in triplicate using the CellTiter-Glo® Luminescent Cell Viability Assay kit (Promega, Madison, WI, USA) according to manufacturer’s instructions and the Trypan-blue assay. The luminescence-based measurements were carried out in a Turner TD20/20 luminometer, with the following setup: delay time 5 s, integration time 20 s, sensitivity 50%. For the Trypan-blue cell viability assay, the medium from Sf21-infected cells was totally removed, the cells stained with Trypan-blue 0.4 % (Invitrogen, Carlsbad, CA, USA) for five minutes, and washed with Phosphate Buffered Saline (PBS, 137mMNaCl, 2.7 mMKCl, 10mM Na_2_HPO_4_, 2mM KH_2_PO_4_, pH 7.4) three times. At least six fields of cells were randomly photographed in a light microscope and used to measure cell death. We used Student’s t-test for statistical analysis to compare two means in pairs.

## Results

### Ba3 sequence and characterization

**Figure 1:**
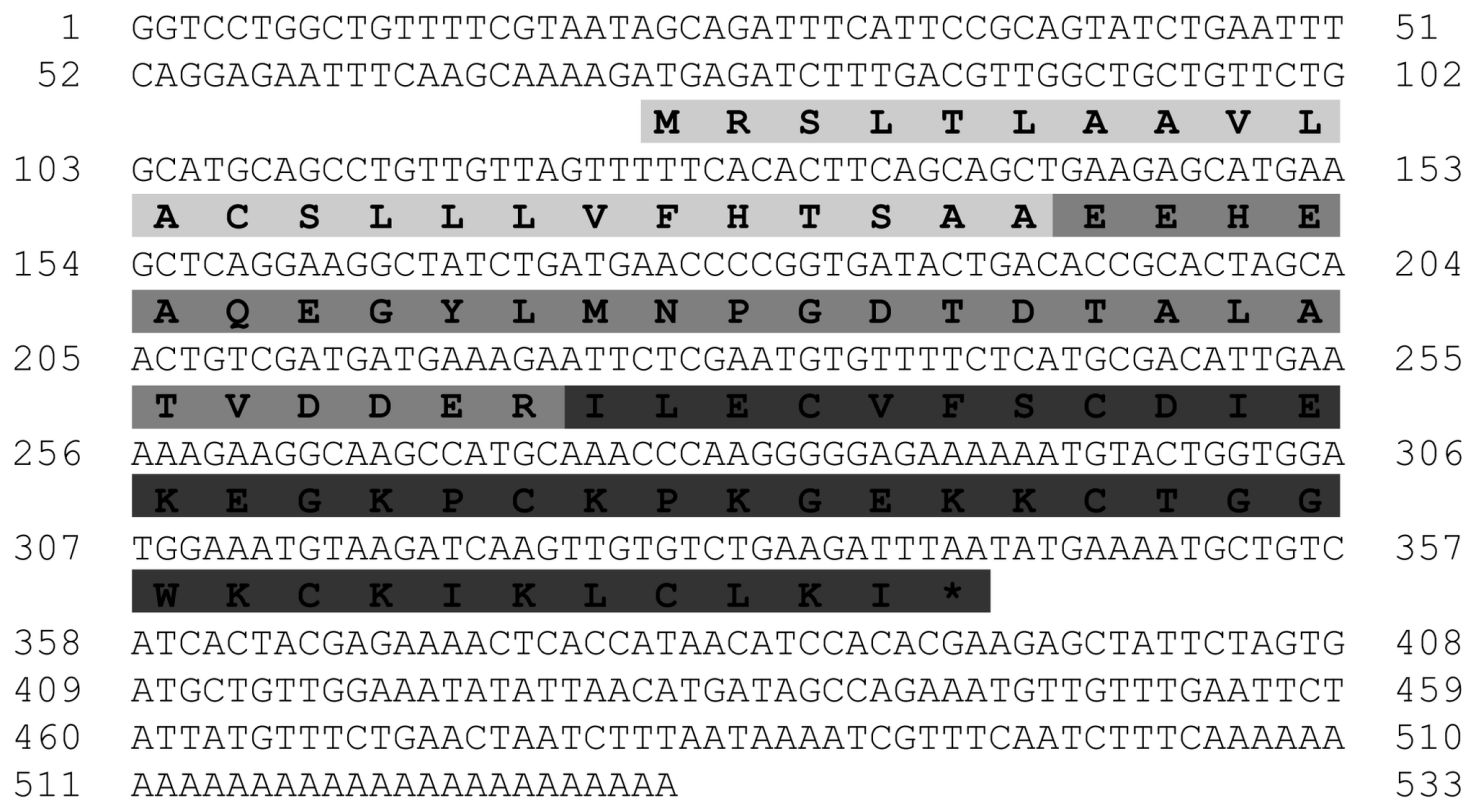
cDNA-derived sequence containing the Ba1 and Ba2-like toxin from the theraphosid *B. albiceps*. The cDNA nucleotide and predicted amino acid sequences of the putative toxin, called here Ba3 (the third toxin purified from *Brachypelma albiceps*, previously known as *B.ruhnaui*) is shown. The signal peptide (light gray), the propeptide (gray), and the mature toxin (dark gray) are highlighted.

The complete sequence of a cDNA clone from the theraphosid *B. albiceps* venom gland was 532 bp long with a 5’-untranslated region (UTR) (72 bp), a putative toxin ORF (270 pb), and a 3’UTR with a poly-adenylation signal 138 bp downstream of the ORF stop codon (190 bp). The coding region was predicted to contain three distinct regions generally observed in arachnid toxin ORFs [35,44] (Figure 1): a signal peptide (SP - 23 amino acids residues, aa) predicted *in silico* for secretion; a propeptide (Pp – 27 aa) with unknown function; and the mature toxin (named as Ba3- 39 aa).

**Figure 2:**
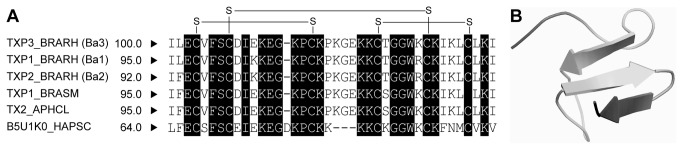
Amino acid sequence and structural prediction of the *B. albiceps* toxin (Ba3). (A) Alignment of the putative mature Ba3 toxin amino acid sequence with related toxins. Gaps have been inserted to achieve best alignment using MAFT method (Material and Methods). Strictly conserved residues have a black background. Moreover, the identity and the entry name in UniProt database are shown. Both TXP1_BRARH (Ba1) (Uniprot: P85497) and TXP2_BRARH (Ba2) (Uniprot: P85504) were toxins previously found within the *B. albiceps* venom extract. TXP1_BRASM (Uniprot: P49265) and TX1_APHCL (Uniprot: P61510) were derived from *B. smithi* and *Aphonopelma californicum*, respectively, and B5U1KO_HAPSC (Uniprot: B5U1K0) was purified from *Haplopelma schmidti*. Furthermore, prediction of the connectivity of the three disulfide bonds is shown for Ba3. (B) Secondary structure was acquired by *in* *silico* analysis showing three strand β-sheet conformation.

The mature form of the Ba3 toxin with 39 aa is proposed based on amino acid alignment with other theraphosid toxins purified from crude venom extracts and available at Uniprot database (TXP1_BRARH (Ba1 - P85497) and TXP2_BRARH (Ba2 - P85504) from *B*. albiceps, TXP1_BRASM (P49265) from *B. smithi*, TX1_APHCL (P61510) from *Aphonopelma californicum*, and B5U1KO_HAPSC (B5U1K0) from the Chinese bird spider *Haplopelma schmidti*) using the MAFFT method [38] (Figure 2A). Interestingly, toxins previously described (Ba1 and Ba2) from *B. albiceps* crude venom showed only a few amino acid differences when compared to Ba3; Ba1 and Ba2 toxins have a Lysine residue at position 11 and an Arginine at position 29, while Ba3 have a Glutamic acid and a Lysine, respectively. Ba2 also have a Phenylalanine at position 2 while Ba3 and Ba2 have a Leucine. The *in silico* analysis showed also that Ba3 mature form has 25% of lysine residues, a three-stranded β-sheet (Figure 2B) with three predicted disulfide-bonds (C4-C17; C8-C31; and C25-C36) (Figure 2A). To avoid misinterpretation, the common name adopted here for the toxin, Ba3, followed the same criteria used for the two previously described toxins from *B. albiceps* venom.

### Construction of recombinant viruses

**Figure 3:**
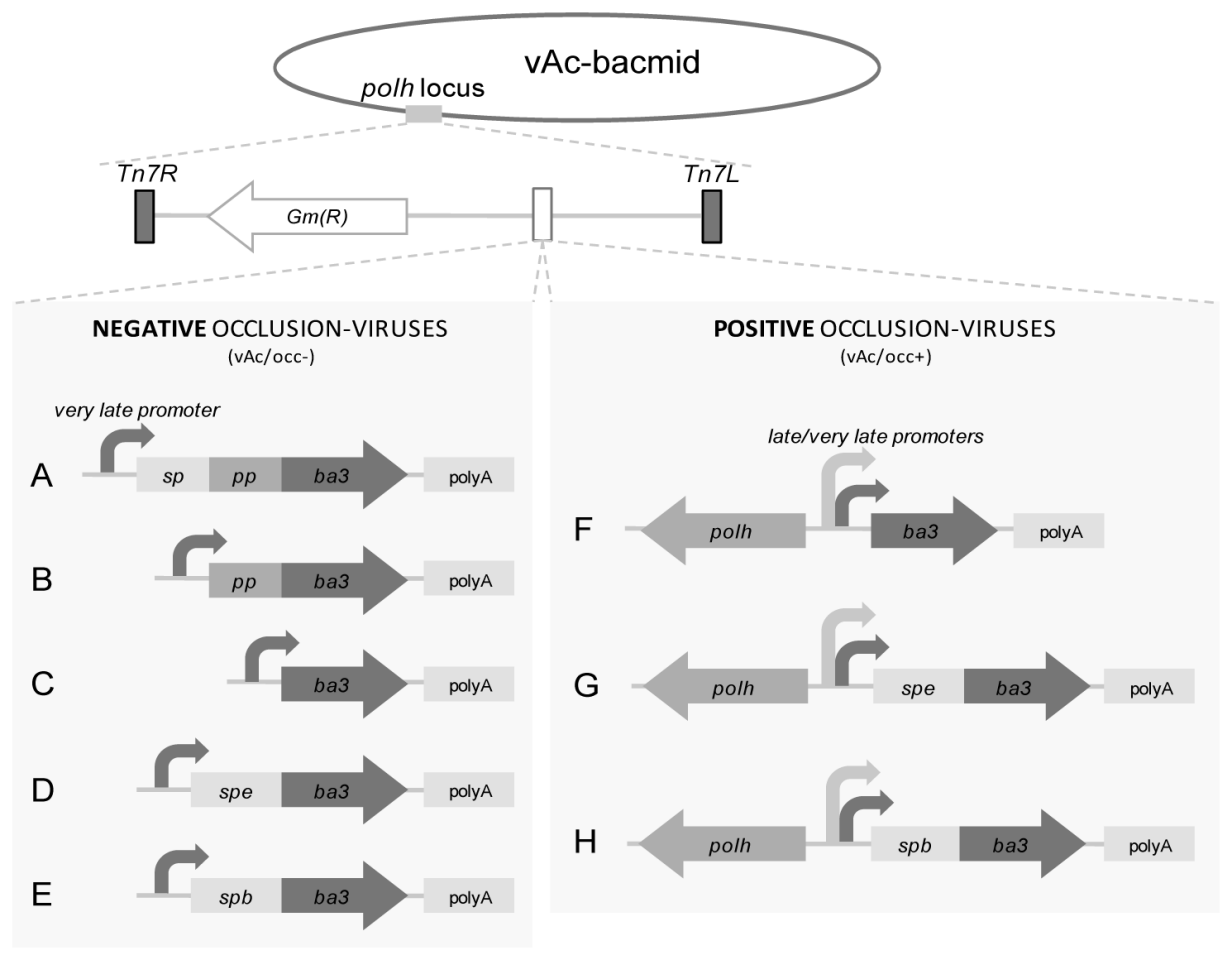
Construction of recombinant bacmids containing different gene variants of the Ba3 toxin. Schematic diagram of occlusion (occ) negative and positive viruses constructed in this work. The variants were inserted into the *polyhedrin* (polh) locus by Tn7-mediated transposition in *E. coli*. Five (A to E) occlusion negative viruses (vAc/occ-) express the toxin gene under a very late (*polyhedrin*) promoter control and three (F to H) occlusion positive viruses (vAc/occ+) are under the control of a late and very late (pSyn and pXIV) promoters *in tandem*. *sp*: signal peptide; *pp*: propeptide; *ba3*: mature toxin; *spe*: signal peptide from a baculoviral gene; *spb*: signal peptide from an insect gene; polyA: poly-adenilation signal.

The different Ba3 toxin gene variants were obtained by PCR, cloned, sequenced, and inserted separately into a commercial transfer plasmid and were then used for the construction of recombinant baculoviruses by transposition in prokaryotic cells (Figure 3). The baculovirus gene expression in infected insect cells occurs in three transcriptional phases: early, late, and very late. The recombinants named vAc/occ- had the toxin under the control of a strong viral promoter active in the very late phase of infection and do not have the *polyhedrin* gene (hence, named occ-, without polyhedra production). Furthermore, we modified this shuttle vector to obtain occluded recombinant viruses expressing the toxin variants; they were named vAc/occ+ (occ+, occlusion positive viruses) and had the variants of the toxin gene under the control of two strong viral promoters active in the late and very late phase of infection.

The occ- baculoviruses containing the pre-propeptide (Figure 3A, *sp-pp-ba3*), propeptide (Figure 3B, *pp-ba3*), mature toxin genes (Figure 3C, *ba3*), and the mature toxin gene in frame with the signal peptides from the AgMNPV*egt* gene (Figure 3D, *spe-ba3*) [45] and the *bombyxin* gene from the silkworm *B. mori* (Figure 3E, *spb-ba3*) [40] were engineered. We also constructed occ+ baculovirus containing the mature toxin gene (Figure 3F, *ba3*) and the mature toxin gene in frame with the signal peptides from the AgMNPV *egt* gene (Figure 3G, *spe-ba3*) and the *bombyxin* gene from the silkworm *B. mori* (Figure 3H, *spb-ba3*).

### Microscopic analysis of cells expressing the mature form of Ba3

**Figure 4:**
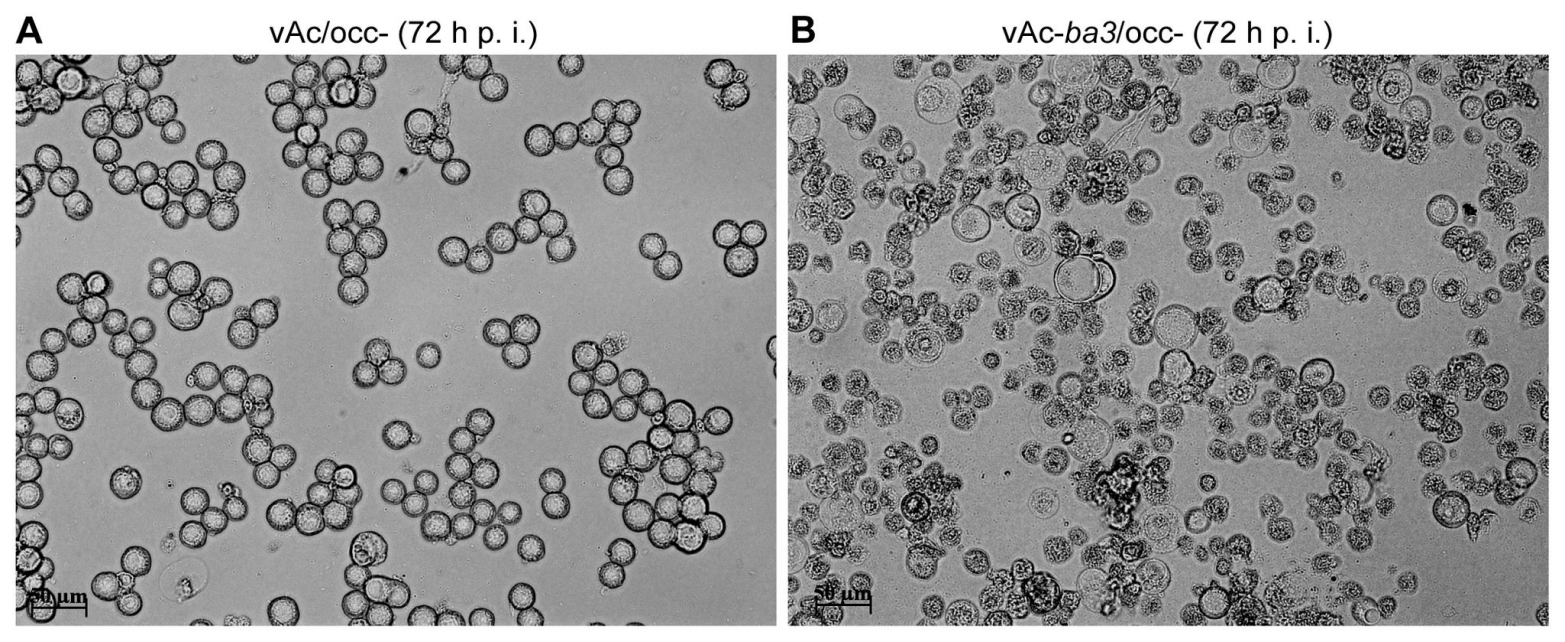
The Ba3 toxin causes cell death by necrosis during recombinant baculovirus infection. Analysis of occlusion negative recombinant baculovirus infection by light microscopy of Sf21 monolayer infected with (A) vAc/occ- (control virus) and (B) the recombinant virus expressing the mature toxin Ba3 (vAc-*ba3*/occ-). In the right panel most of the cells are dead. Photographs were taken 72 h after virus infection.

**Figure 5:**
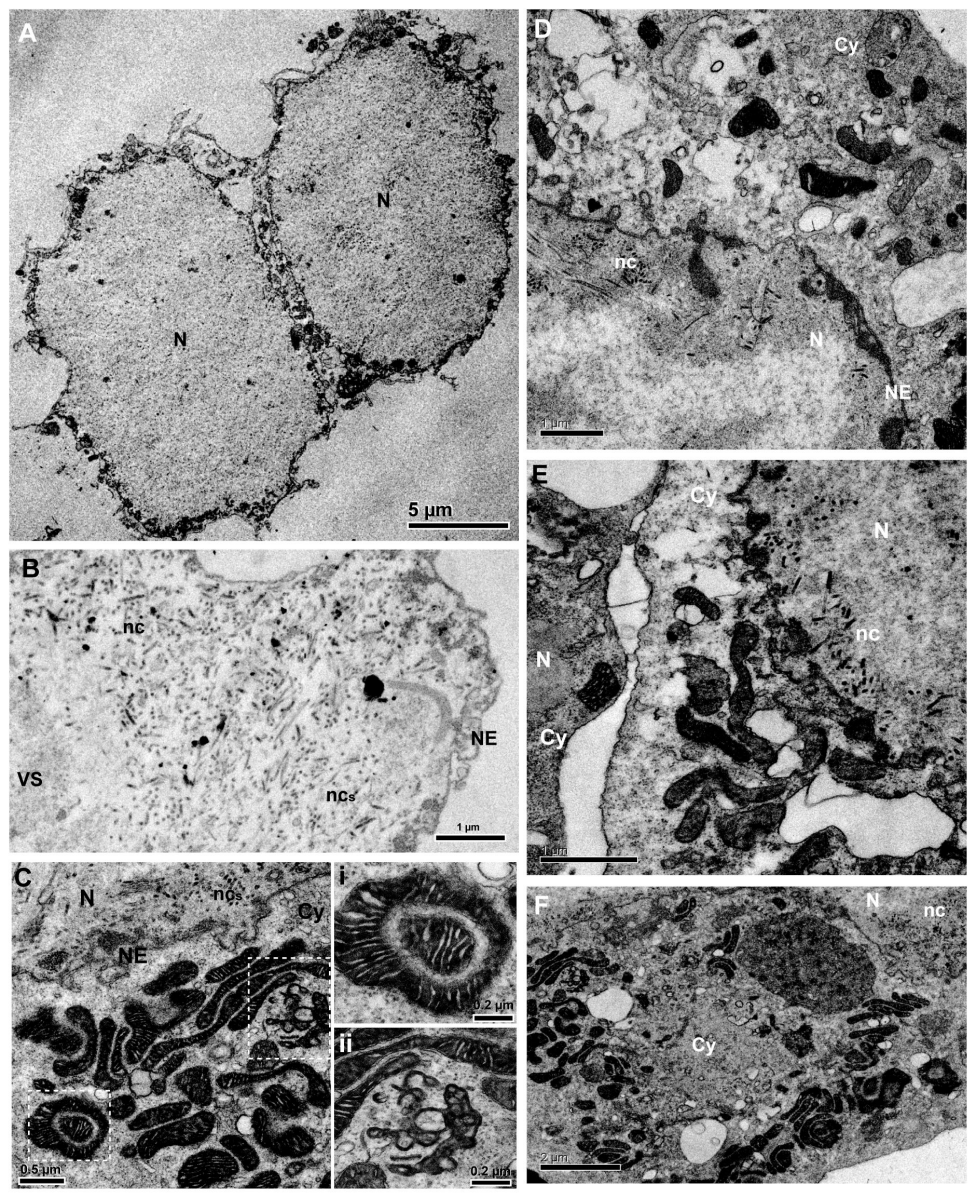
Ultrastructural effects of Ba3 toxin expression in recombinant virus-infected Sf21 cells. Transmission electron microscopy analysis of Sf21 cells infected with vAc-*ba3*/occ-. (A) Cells infected (36 h p.i.) with the recombinant virus expressing the mature toxin showing absence of cytoplasm. (B) Cell nucleus (N) showing rod-shaped nucleocapsids (ncs) close to the nuclear envelope (NE). (C) Changes in mitochondrial morphology prior to cell death (36 h p.i.) in Sf21-infected cells. Insets (i and ii) show altered mitochondria. (D) Cell infected (36 h p.i.) with the recombinant control virus (vAc/occ-).. (E and F) Cells infected (36 h p.i.) with the recombinant virus expressing the mature variant of the spider toxin Ba3. The main difference observed at 36 h p.i. is the presence of altered mitochondria in cells infected with the recombinant virus containing the mature form of the Ba3 toxin gene. These conclusions are based on several observed cells and were not observed in cells mock-infected (data not shown).

For light and transmission electron microscopy, Sf21 cells were infected with vAc-*ba3*/occ- and vAc/occ- (moi of 10) and the cells were analyzed at various time intervals p.i. Until 24 h p.i., the recombinant virus containing the mature toxin gene under the control of a very late promoter (vAc-*ba3*/occ-) showed the same cytomorphological changes as the control virus (vAc/occ-) such as nuclear hypertrophy and cell rounding [46] (Figure S1). However, at 24 h p.i. we observed increased cell death in cells infected with the recombinant virus relative to the control virus (Figure 4A and B). Under light microscopy, dead cells appeared smaller than live cells and apparently lacked cytoplasm. Dead cells were always observed in monolayers infected with all recombinant viruses tested harboring different variants of the toxin, but were present in different amounts depending on the infecting virus (Figure S2, arrows). To further investigate these cells, they were infected with vAc-*ba3*/occ- and vAc/occ- and were processed at 36 hp.i. for TEM analysis (Figure 5). Dead cells showed viral nucleocapsids inside the nucleus (Figure 5A and B) and no cytoplasm (Figure 5A). We also observed increased numbers of mitochondira altered morphologically in cells apparently live (Figure 5C and E-G). Organelles ring-shaped and C-shaped were observed and many mitochondria seemed to contain ball-like structures (Figure 5C, panel i) and had internal lumen surrounded by membranes. The lumen of some organelles connected to the external cytoplasm was fragmented (Figure 5C, panel ii). None of these features were observed in cells infected with the control virus (Figure 5D).

### Cytotoxicity assays

**Figure 6:**
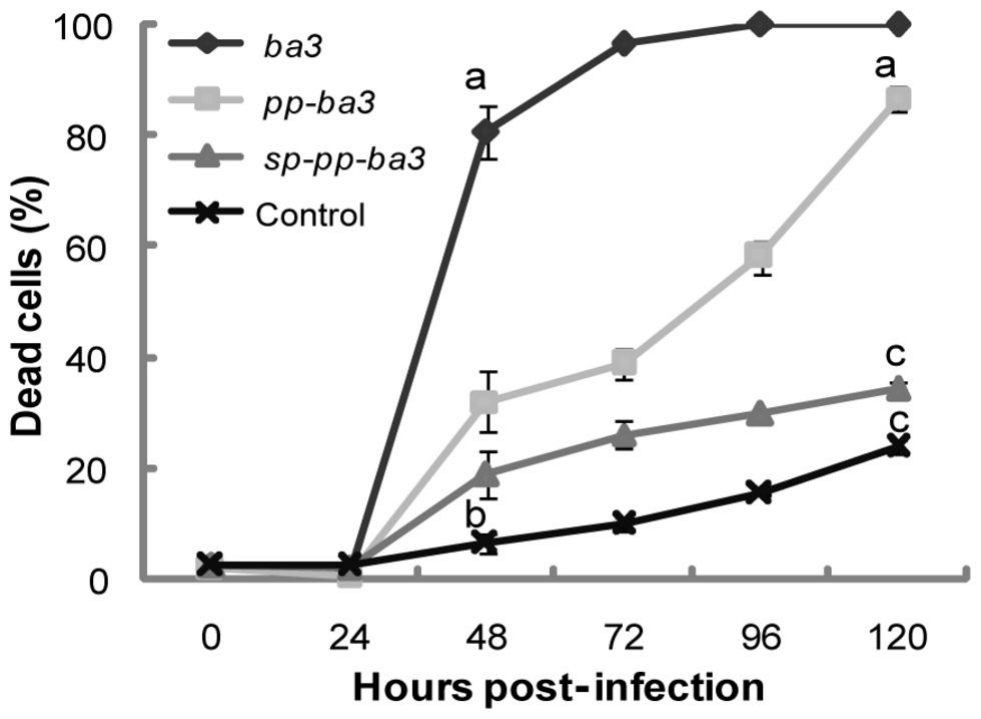
Cell viability quantification during negative-occlusion recombinant baculovirus infection. Recombinant viruses containing the mature toxin gene (diamond - *ba3*), the propeptide (square - *pp*-*ba3*), the pre-propeptide (triangle - *sp*-*pp*-*ba3*), or no toxin (x - Control) during infection of Sf21 cells at the indicated times post-infection (h p.i.). The results represent averages of at least three independent experiments observed at different times post-infection, and standard errors are indicated. The letters a, b, and c indicate punctual significant or not significant differences by Student’s t-test (a/b *P*<0.001; b/c *P*<0.005; c/a *P*<0.001).

**Figure 7:**
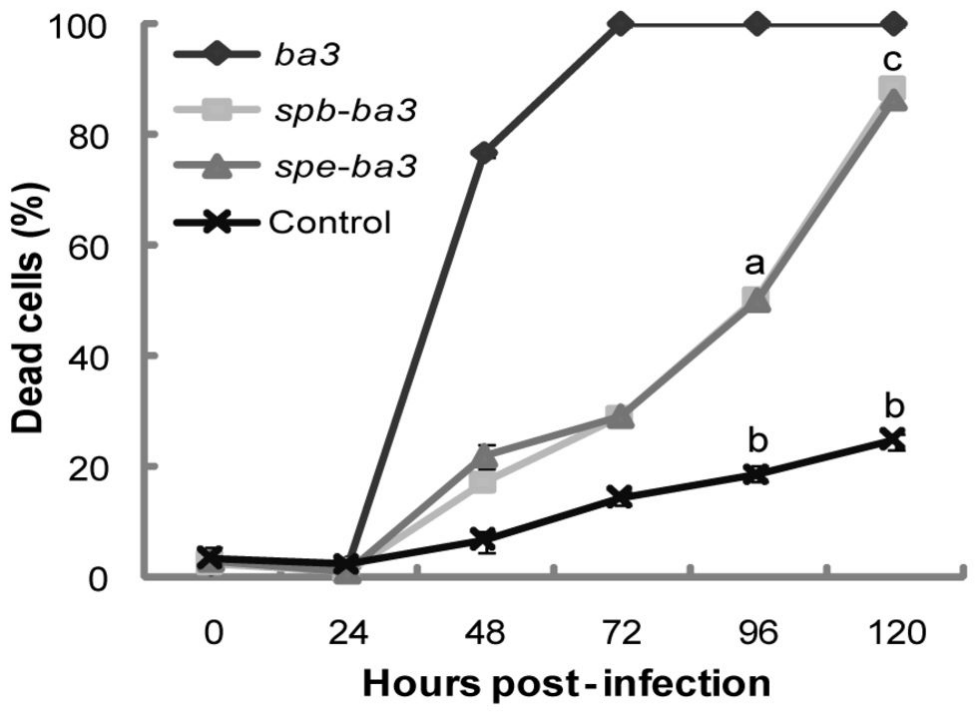
Cell viability quantification during positive-occlusion recombinant baculovirus infection. The recombinant virus containing the mature toxin gene (diamond - *ba3*), the toxin gene fused in frame to a signal peptide derived from an insect gene (square - *spb-ba3*) or from a baculovirus gene (triangle - *spe-ba3*), or no toxin (x) during infection of Sf21 cells at the indicated times post-infection (h p.i.). The results represent averages of at least three independent experiments observed at different times post-infection, and standard errors are indicated. The letters a, b, and c indicate punctual significant or not significant differences by Student’s t-test (a/b *P*<0.0001; b/c *P*<0.001; c/a *P*<0.0001).

Sf21 cells were separately infected with each of the recombinant viruses (moi of 10),and cell viability was measured. We tried to use a commercial viability assay kit (CellTiter-Glo® Luminescent Cell Viability Assay kit from Promega, Madison, WI, USA) based on ATP level available into the cell or released by cell death. Luciferase activity is ATP-dependent and we expected to be able to measure death using this assay since we have previously used this kit with success to access cell death induced by *Bacillus thuringiensis* Cry toxins in insect and mammalian cell lines [47]. However, we only obtained arbitrary values for light unit measurements during recombinant virus infection using cell extracts and supernatants (data not shown). Therefore, we used the Trypan-blue method at different times p.i. to detect cell death in Sf21 cells infected with different recombinant viruses. All toxin variants expressed by recombinant baculovirus in insect cells caused widespread death by necrosis when compared to the control (Figure 6). Dead cells increased during infection due to the fact that the gene was under the control of a very late promoter. The recombinant virus expressing the mature form of the toxin gene (vAc-*ba3*/occ-) induced death in 80 % (± 4.8%) of the cells by 48 h p.i., while for the control virus (vAc/occ-) only 6.5 % (± 1.5%) (*P*< 0.001) were dead at this time p.i. (Figure 6). Interestingly, we found that the recombinant virus containing the protoxin gene (vAc-*pp-ba3*/occ-) had a delayed effect on cell death compared to vAc-*ba3*/occ-, reaching 96.8 % (± 2.7 %) only at 120 h p.i.(Figure 6). There was little difference between the recombinant virus containing the pre-propeptide gene (Ac-*sp-pp-ba3*/occ-) and the control vAc/occ-, and no significant difference even at 120 h p.i. (Figure 6). The other recombinant viruses containing the mature toxin gene fused with the baculovirus *egt* signal peptide (vAc-*spe-ba3*/occ-) or the signal peptide for the *B. mori bombyxin* (vAc-*spb-ba3*/occ-) did not cause any significant alteration in cell viability as did those observed for the mature form and the propeptide form (compare Figure S3A and Figure 6). In contrast, we observed that the different promoters used to drive expression of the *spe*-*ba3* and *spb*-*ba3* variants altered the amount of dead cells (Figure 7 and S3). The mature variant *ba3* expressed by composite late and very late promoter (Figure 3F) induced 100% of cell death earlier (72 h p.i., Figure 7) than when expressed under only the control of the very late promoter of *polyhedrin* (96 h p.i., Figure S5) only in Tn5B cells.

### Polyhedra production by the recombinant occ+ virus

There were no OBs produced by occlusion positive recombinant viruses containing the mature toxin gene (*ba3*) or the toxin gene fused to insect- or baculovirus-derived signal peptide (*spb-ba3*and *spe-ba3*) during *in vitro* (Figure S4) or *in vivo* (Table 1) infection where as the control virus (vAc/occ+) did.

**Table 1:** Polyhedra and polyhedra-like structures recovered from 1 g of dead *Spodoptera frugiperda*l arvae by infection with different recombinant viruses.

Virus	Polyhedra	S.D.**^*1*^**
vAc/occ+	2 x 10^6^	± 2.06 x 10^5^
vAc-*ba3*/occ+	66.6	± 65.06
vAc-*spe-ba3*/occ+	28.3	± 15.6
vAc-*spb-ba3*/occ+	16.3	± 8.1

^1^ = standard deviation

## Discussion

A newly discovered putative spider toxin gene *ba3* was found in a cDNA library derived from from the Mexican golden redrump theraphosid *Brachypelma albiceps* venom gland. The deduced amino acid sequence of Ba3 was found to be closely related to the Ba1 and Ba2 toxins (Figure 2), two insect specific peptide toxins that lack toxicity in mice and were previously purified from the venom of the same spider [33]. Ba1 and Ba2 present insecticidal activity against house crickets but the cellular targets and mode of action of both are still not known. The Ba3 structural prediction indicates it contains a three-stranded β-sheet with three disulfide-bonds (C4-C17; C8-C31; and C25-C36), which could suggest classification of the Ba3 conformation as a “Disulfide Directed Beta-Hairpin” motif (DDH) whose consensus sequence proposed by Wang et al. [48] is CX_5−19_CX_2_(G or P)X_2_CX_6−19_C.

To understand Ba3 function as an insecticidal peptide for biological control, we first performed *in silico* analyses which indicated that Ba3 and its paralogous toxin peptides are potential cytotoxins. Kourie and Shorthouse [49] have defined one class of cytotoxic peptides as small cationic proteins with three or four disulfide-bonds, and are composed of only beta-sheets as was predicted for Ba3 (Figure 2). Cytotoxins are capable of forming direct or indirect (by ionic channels interaction) membrane pores, and also, interfere with signal transduction and homeostasis that kill the cell [50–53]. Several cytotoxic molecules have been found in spider venoms [54–58]. Cohen and Quistad [59] tested venom extracts from several arthropods against different cell lines such as *S. frugiperda*-derived Sf9 cells. Among the venoms tested, two were from spiders of the genus *Aphonopelma* (non identified at species level). Surprisingly, the venom of both spiders showed toxic activity against *S. frugiperda* cells. *Brachypelma* and *Aphonopelma* genera are theraphosids phylogenetically related, and until 1997, *B. ruhnaui*, and now *B. albiceps*, was classified in the genus *Aphonopelma* [60].

The experimental results shown in this work, such as morphological changes evident from light (Figure 4) and transmission electron microscopy (Figure 5), and cytotoxicity (Figures 6 and 7) suggest that Ba3 was able to kill insect cells by necrosis when expressed by and associated to baculovirus infection. Therefore, baculoviruses expression vectors harboring spider-specific peptide toxins are potential tools for studying at cellular level the mode of action of insecticidal toxins having unknown functions, besides the virus effects on the cell must be considered.

The Committee on Cell Death recognizes major different types of cell death characterized by morphological and molecular criteria; one of these is necrosis [61]. Characteristically, this type of cell death has been considered disordered and merely accidental cell death, and was defined by the absence of hallmarks of apoptosis or autophagy but with loss of the cell membrane integrity to physical, chemical, biochemical, or pathological damages [62] being able also to occur in a regulated manner [63].

The Trypan-blue dye exclusion assay employed to quantify cell death has been widely used to analyze insect cell death [64–67]. Moreover, our conclusion is not solely based on this experiment, but was also confirmed by the structural and ultra-structural changes observed during toxin expression in *S. frugiperda* cells (Figure 4 B and 5). The Trypan-blue dye exclusion assay may in fact overestimate cell viability [68]. We have also evaluated cytotoxicity during recombinant virus infection of Tn5B cells with all baculoviruses expressing the toxin variants and found similar results when compared to Sf21 cells (Figure S5A and B). However, we did observe differences in the time when cell death occurred in the two cell lines. Tn5B cells died earlier (24 h p.i.) despite being more resistant to infection with vAc-*ba3*/occ- than Sf21 (at 48 h p.i. Tn5B presented 40.16 ± 1.13 % of dead cells while Sf21 presented 80.71 ± 4.78 %, with *P*< 0.001). Interestingly, *S. frugiperda* cells are less efficient than Tn5B cells for recombinant protein production from *polyhedrin* promoter during engineering AcMNPV infection [69–71]. Tn5B cells probably have had elevated mortality earlier in infection because their better efficiency in expressing recombinant proteins, regardless of their higher resistance to death as was previously shown [72]. An untested assumption is that Sf21 cells are more sensitive to the toxin, but are less efficient in expressing the recombinant peptide at the earlier times post infection.

We found that the expression of the Ba3 toxin with immature regions such as signal peptide and propeptide during infection of insect cells clearly delayed cell death (Figures 6 and 7).The length and amino acid composition of signal peptides and propeptides are quite variable and are essential for expression and processing of resident or organelle proteins [73]. When heterologously expressed, both regions sometimes remain in the protein, indicating a lack of processing capability by the host cell. Tessier et al. [74] observed accumulation of pre-propapain in the cytoplasm of insect cells infected by recombinant baculovirus. To circumvent this problem, they fused the protein to an insect-derived signal peptide which resulted in increased protein secretion. However, the change or adequacy for signal peptide to the target expressed protein does not guarantee the correct protein processing and hence the incursion in the exocytic pathway as previously observed [75–77]. Although an evolutionary relationship between insects and arachnids exist, there is no guarantee that the spider signal peptide or pro region of propeptide will be recognized and/or correctly processed by the virus-infected insect cell protein machinery [78,79].

We showed that different levels of cell death occurred during infection of insect cells with recombinant viruses containing the mature toxin fused to signal peptides with high probability to be recognized by the insect cell protein mechanisms or fused to the spider immature region (Figure 7). As previously observed for Bombyxin and EGT derived-signal peptides, the choice of signal sequence fused with an insect toxin resulted in significant effects for the virus pathology, mainly if the expression is controlled by an early promoter in insects [78,80]. Since the different *ba3* gene variants were under the control of active late promoters, the difference in cell death between the immature and mature forms of the toxin was probably due to the compromising of the protein expression and secretion caused by the late viral infection. Moreover, Chejanovsky et al. [28] showed that insect cells were able to express, secrete, and process correctly the recombinant peptide when infected with a recombinant baculovirus, containing the scorpion toxin LqhαIT gene fused to same signal peptides (*spe* and *spb*).

Since we could not quantify infected-cell death by the CellTiter-Glo® Luminescent Cell Viability Assay kit based on ATP levels in cells or that is released due to cell lysis, we investigated the cytomorphological changes that occurred prior to cell death. It is known that mitochondria are responsible for ATP production and control of cell death (i.e. apoptosis) which requires evolutionary conserved mechanisms related to cytochrome c and mitochondrial pro-apoptotic proteins [81]. However, apoptosis is not the unique cell death mechanism linked to mitochondria control. For example, hyperpolarization of the organelle membrane potential can induce oxidative stress and cell death by necrosis in human cells [82]. In our experiments, the presence of the mature toxin form of Ba3 gene in the recombinant baculovirus altered the insect cell mitochondria morphology prior to cell death by necrosis (Figure 5C and E-G). Importantly, all of the morphological changes observed in mitochondria by transmission electron microscopy resembled structures previously described for murine mitochondria in response to oxidative damage [83] or high doses of arthropod venom [84]. We hypothesized that the toxin might act directly or indirectly on some receptor of mitochondria, altering its natural operation and, as a consequence, causing insect cell death by necrosis. Mitochondria are the most important organelles for ATP production, muscle and cell performance, and control of cell death. Thus under ecological sights, could be important target for predator toxin action. Interestingly, Siemens et al. [85] found three inhibitor cysteine knot peptides non-related to Ba3 in the venom from a tarantula, that target transient receptor potential channels (TRP-channels) to produce inflammatory pain in mammalian. These channels are present at several regions in cells such as at the plasma membrane, in the endoplasmic reticulum, endosomes, Golgi, nuclei, peroxysomes, and mitochondria [86], but its function remains unclear. Generalist receptors like TRP-channels can be interesting targets for spider toxin action [87].

Despite our efforts, we were unable to detect the Ba3 toxin peptide by immunolabelling using two different strategies for antibody production (data not shown). Firstly, we fused a hexa-histidine tag (using the pDEST17 vector from Invitrogen, Carlsbad, CA, USA) to the amino-terminus end of the peptide in the *ba3* form. Despite the plasmid sequence being confirmed by sequencing (Macrogen, Seoul, Korea) and the bacterial transformation by PCR, the Ba3 peptide expressed in *E. coli* strain BL21-AI (Invitrogen, Carlsbad, CA, USA) was not detected by neither affinity chromatography experiments nor Western Blot (anti-hexa-histidine antibody). Furthermore, another *E. coli* strain (Rosetta-gami – Novagen) was tested; however, no recombinant colonies were observed (data not shown). These strains are recommended for toxic protein expression and, in the case of Rosetta-gami, there is a propitious cell environment for disulfide-bonds-rich protein expression. Several recommended protocols for expression of toxic proteins were tested but no colonies were recovered. We assumed that these results were probably due to some intrinsic feature of the toxin, such as the cytotoxic effect found in this work for insect cells. Secondly, we fused the toxin gene to the carboxi-terminus of the glutathione s-transferase gene (using the pDEST15 vector from Invitrogen, Carlsbad, CA, USA) (data not shown). The recombinant protein was purified and confirmed by Western Blot. Surprisingly, rats and mice immunized with GST-Ba3 only produced antibody against GST probably due to the small Ba3 peptide size.

The use of a protein tag for peptide immunodetection might not be a good strategy for protein detection due to the fact that this region could influence on the small peptide function biasing the results. A previous study using the spider Alpha-latrotoxin, a potent stimulator of exocytosis from neurons and neuroendocrine mamalliang cells that consists of a conserved N-terminal domain and C-terminal ankyrin-like repeats, showed that the tag-fused toxin version was much less potent compared to the untagged one [88]. Thus, we assumed the presence of the toxin during insect cell baculovirus infection based on indirect features observed during cell infection, such as cytotoxicity, structural and ultrastructural analysis, since these features were not observed for the control virus without the toxin versions. A previous work also assumed the presence of a scorpion toxin expressed by baculovirus based on larval behavior during infection [80]. The differences in cytotoxicity of the constructs could be due to variation in toxin expression levels rather than to different efficiency of toxin action. However, the same number of virus (moi) was used per cell and also all the toxin versions were under the control of the same promoter. Therefore, whether the variation in the toxin expression level exists it must be solely restricted to features of the toxin gene since it was the only factor that varied in the experiment. Importantly, we carried out time point analysis for the total mRNA produced by Sf21 cells during recombinant virus infection for all toxin versions in order to identify the toxin transcription. We were able to identify the toxin transcripts in all versions by RT-PCR (data not shown). However, this technique and also RT-qPCR is not recommended for baculovirus transcriptional analysis due to intrinsic features of the virus, such as, extension of the transcript ends to neighbour genes [89].

The expression of the toxin during baculovirus infection did not block budded virus production during the course of infection. We observed virus nucleocapsids within the nucleus of dead cells (Figure 5) and also no difference in the virus titer (data not shown). This fact can be explained mostly by the fact that the toxin versions were under control of a very late promoter, and the budded virus production occurs prior to the massive activation of this promoter [90] We also detected no polyhedra production during *in vitro* (Figure S4) and *in vivo* (Table 1) infection with the occlusion positive recombinant baculoviruses. In this case, the premature cell death probably prevented accumulation of Polyhedrin resulting in the nucleus and polyhedra production. Indeed, Jarvis et al. [76] showed that the Polyhedrin nuclear localization efficiency depends on its biosynthesis ratio, which can explain the absence of nuclear occlusion body during infection. 

Insecticidal toxin expression by recombinant baculoviruses in insect cells can be used to understand the biological function of unknown peptides from arthropods at the cell or insect levels. This is the first report showing the cytopathic effects caused by engineered baculovirus containing a putative cytotoxic peptide gene and is also the first reported recombinant baculovirus harboring a theraphosid-derived toxin. Previous reports with the expression of insecticidal toxins using baculovirus/insect cell system studied the *in vivo* aspects, such as lethal virus doses and larval behavior changes, different from the aspects addressed by this work.

## Supporting Information

Table S1 **Primers used for gene amplification in this work.**
(DOCX)

Figure S1 **Structural analysis of Sf21 cells infected with recombinant and control viruses at 24 h p.i.** Analysis of occlusion negative recombinant baculovirus infection (24 h p.i.) by light microscopy of Sf21 monolayers infected with (A) vAc/occ- (control virus) and (B) the recombinant virus expressing the mature toxin Ba3, (vAc-*ba3*/occ-). No difference structural difference was observed.
(TIF)

Figure S2 **Structural analysis of Sf21 cells infected with recombinant viruses containing the full-length (*sp-pp-ba3*) and a truncated version (*pp-ba3*) of the Ba3 gene.** Analysis of occlusion negative recombinant baculovirus infection by light microscopy of Sf21 monolayers infected with (A) the virus expressing the propeptide variant vAc-*pp-ba3*/occ- and (B) the virus expressing the pre-propeptide variant vAc-*sp-pp-ba3*/occ-. Dead cells are indicated by black arrows. Photographs were taken 72 h after virus infection. The recombinant virus containing the pre-propeptide version of the toxin caused more cell death than the recombinant virus containing the propeptide version (compare A and B).
(TIF)

Figure S3 **Cell viability quantification during negative-occlusion recombinant viruses infection expressing the toxin in frame with different signal peptides.** The recombinant virus containing the mature toxin gene fused in frame to a signal peptide derived from an insect gene (diamond - *spb-ba3*) or from a baculovirus gene (square - *spe-ba3*), or no toxin (x) during infectionof Sf21 and Tn5B cells at the indicated times post-infection (h p.i.). The results represent averages of at least three independent experiments observed at different times post-infection, and standard errors are indicated.
(TIF)

Figure S4 **The Ba3 toxin causes early cell death by necrosis during recombinant baculovirus infections in Tn5B cells.** Analysis of occlusion positive recombinant baculovirus infection by light microscopy of Tn5B monolayers infected with (A) vAc/occ+ (control virus), (B) the recombinant virus expressing the mature toxin Ba3, (vAc-*ba3*/occ+), (C) the recombinant virus expressing the mature toxin Ba3fused in frame with the *bombyxin* signal peptide (vAc-*spb-ba3*/occ+), or (D) with the *egt* signal peptide (vAc-*spe-ba3*/occ+). Even being occlusion positive, the recombinant viruses expressing the different variants of the toxin were not able to form polyhedra inside the nucleus as the control was (black arrows indicate polyhedra accumulation inside the cell nucleus).
(TIF)

Figure S5 **Cell viability quantification during infection of Tn5B cells with negative- and positive-occlusion recombinant baculovirus.** (A) The recombinant virus containing the mature toxin gene (diamond –*ba3*), the propeptide (square - *pp-ba3*), the pre-propeptide (triangle - *sp-pp-ba3*) under the control of a very late promoter, or no toxin (x - Control) during infection of Tn5B cells at the indicated times post-infection (h p.i.). (B) The recombinant virus containing the mature toxin gene (diamond –*ba3*), the toxin gene fused in frame to a signal peptide derived from an insect gene (square - *spb-ba3*), or from a baculovirus gene (triangle - *spe-ba3*), all under the control of a late and very late promoter, or no toxin (x) during infection of Tn5B cells at the indicated times p.i. The results represent averages of at least three independent experiments observed at different times post-infection, and standard errors are indicated. The expression of the different version of the toxin under the control of two promoters (B) induced earlier cell death than the expression of the same toxins under the control of a very late promoter (A).
(TIF)
