# Supplementary material for: A New theraphosid Spider Toxin Causes Early Insect Cell Death by Necrosis When Expressed In Vitro during Recombinant Baculovirus Infection
Source: PLoS One. 2013 Dec 13;8(12):e84404. doi: 10.1371/journal.pone.0084404 (PMC3862797; doi:10.1371/journal.pone.0084404)
Supplement: Table S1 — Primers used for gene amplification in this work. (DOCX) [file pone.0084404.s001.docx]

| Primer name | Sequence |
| --- | --- |
|  |  |
| FA | ATTCTCGAATGTGTTTTCTCATG |
| CDS3 | AATCTTCAGACACAACTTGATC |
| F1 | CGGATCCATGAGATCTTTGACGTTGGCTGCTG |
| F2 | CGGATCC*ATG*GAAGAGCATGAAGCTCAGGAAGGC |
| F3 | CGGATCC*ATG*ATTCTCGAATGTGTTTTCTCATGCGAC |
| F4 | GGATCCatgatttttattttactaacaacgcttttagcagtaagcggagcaaaaaccgcaaatATTCTCGAATGTGTTTTCTCA |
| F5 | GGATCCatgaagatcctccttgctattgcccttatgcttagcaccgtgatgtgggtgagcaccATTCTCGAATGTGTTTTCTCA |
| R | AAGCTTGAAAGATTGAAACGATTTTATTAAAG |
| pSyn F | GCGGCCGCGAATTCGATGATCTATTTATAGG |
| pSyn R | GTCACTAGTTAAATCAACAACGCACAGAATCTAGCGC |
|  |  |
|  | |
| **Underlined**: Restriction sites used for cloning procedures  **Italic**: Unnatural start codon  **Low case**: Signal peptide from insect- or baculovirus-derived gene | |
