# Supplementary figures and images for: A New theraphosid Spider Toxin Causes Early Insect Cell Death by Necrosis When Expressed In Vitro during Recombinant Baculovirus Infection
Source: PLoS One. 2013 Dec 13;8(12):e84404. doi: 10.1371/journal.pone.0084404 (PMC3862797; doi:10.1371/journal.pone.0084404)

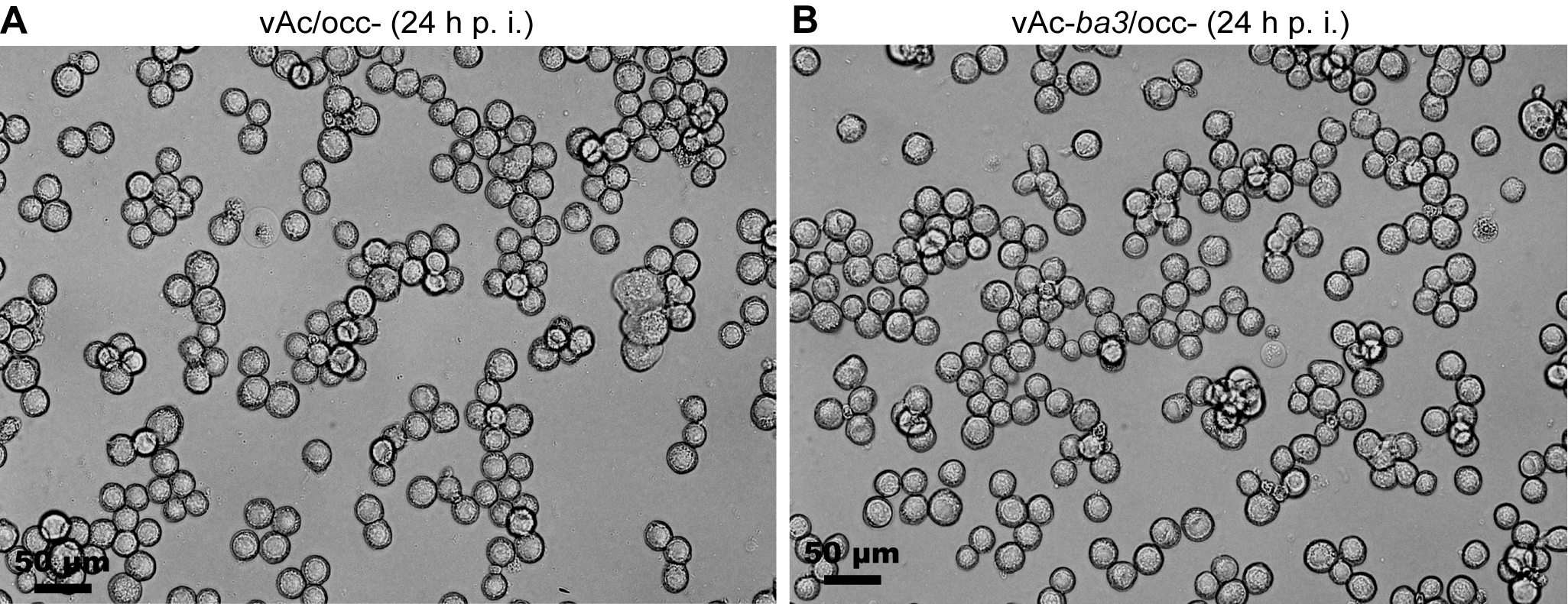

Supplement: Figure S1 — Structural analysis of Sf21 cells infected with recombinant and control viruses at 24 h p.i. Analysis of occlusion negative recombinant baculovirus infection (24 h p.i.) by light microscopy of Sf21 monolayers infected with (A) vAc/occ- (control virus) and (B) the recombinant virus expressing the mature toxin Ba3, (vAc-ba3/occ-). No difference structural difference was observed. (TIF) [file pone.0084404.s002.tif]

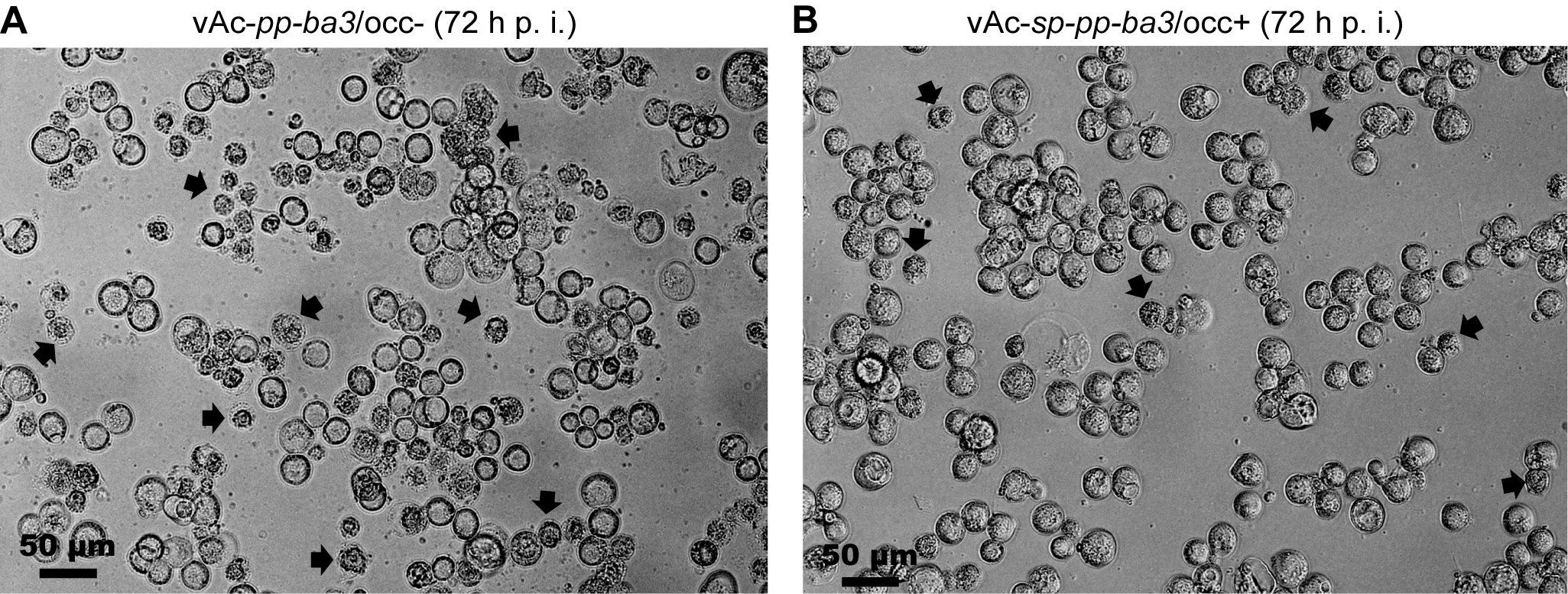

Supplement: Figure S2 — Structural analysis of Sf21 cells infected with recombinant viruses containing the full-length (sp-pp-ba3) and a truncated version (pp-ba3) of the Ba3 gene. Analysis of occlusion negative recombinant baculovirus infection by light microscopy of Sf21 monolayers infected with (A) the virus expressing the propeptide variant vAc-pp-ba3/occ- and (B) the virus expressing the pre-propeptide variant vAc-sp-pp-ba3/occ-. Dead cells are indicated by black arrows. Photographs were taken 72 h after virus infection. The recombinant virus containing the pre-propeptide version of the toxin caused more cell death than the recombinant virus containing the propeptide version (compare A and B). (TIF) [file pone.0084404.s003.tif]

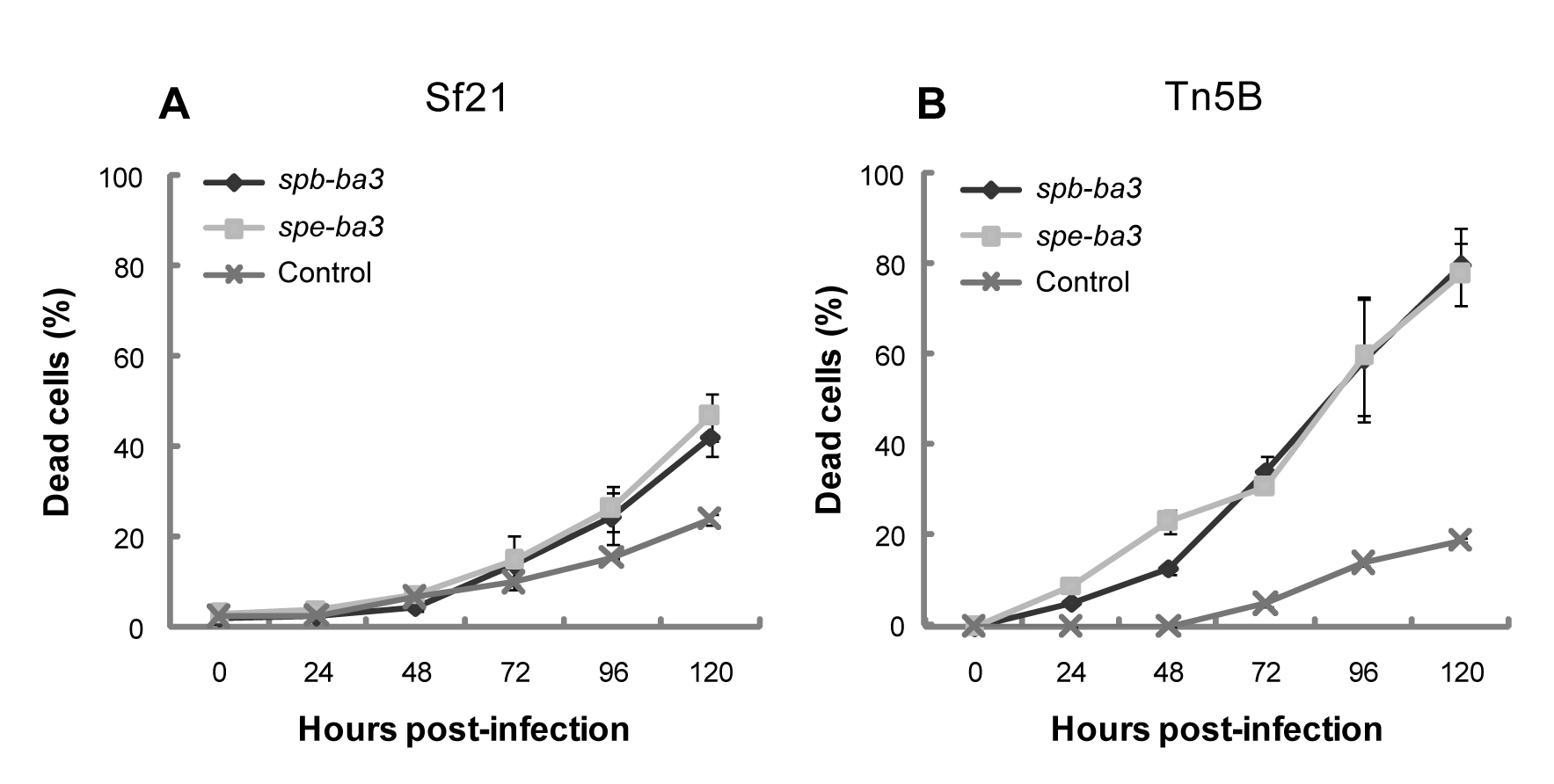

Supplement: Figure S3 — Cell viability quantification during negative-occlusion recombinant viruses infection expressing the toxin in frame with different signal peptides. The recombinant virus containing the mature toxin gene fused in frame to a signal peptide derived from an insect gene (diamond - spb-ba3) or from a baculovirus gene (square - spe-ba3), or no toxin (x) during infectionof Sf21 and Tn5B cells at the indicated times post-infection (h p.i.). The results represent averages of at least three independent experiments observed at different times post-infection, and standard errors are indicated. (TIF) [file pone.0084404.s004.tif]

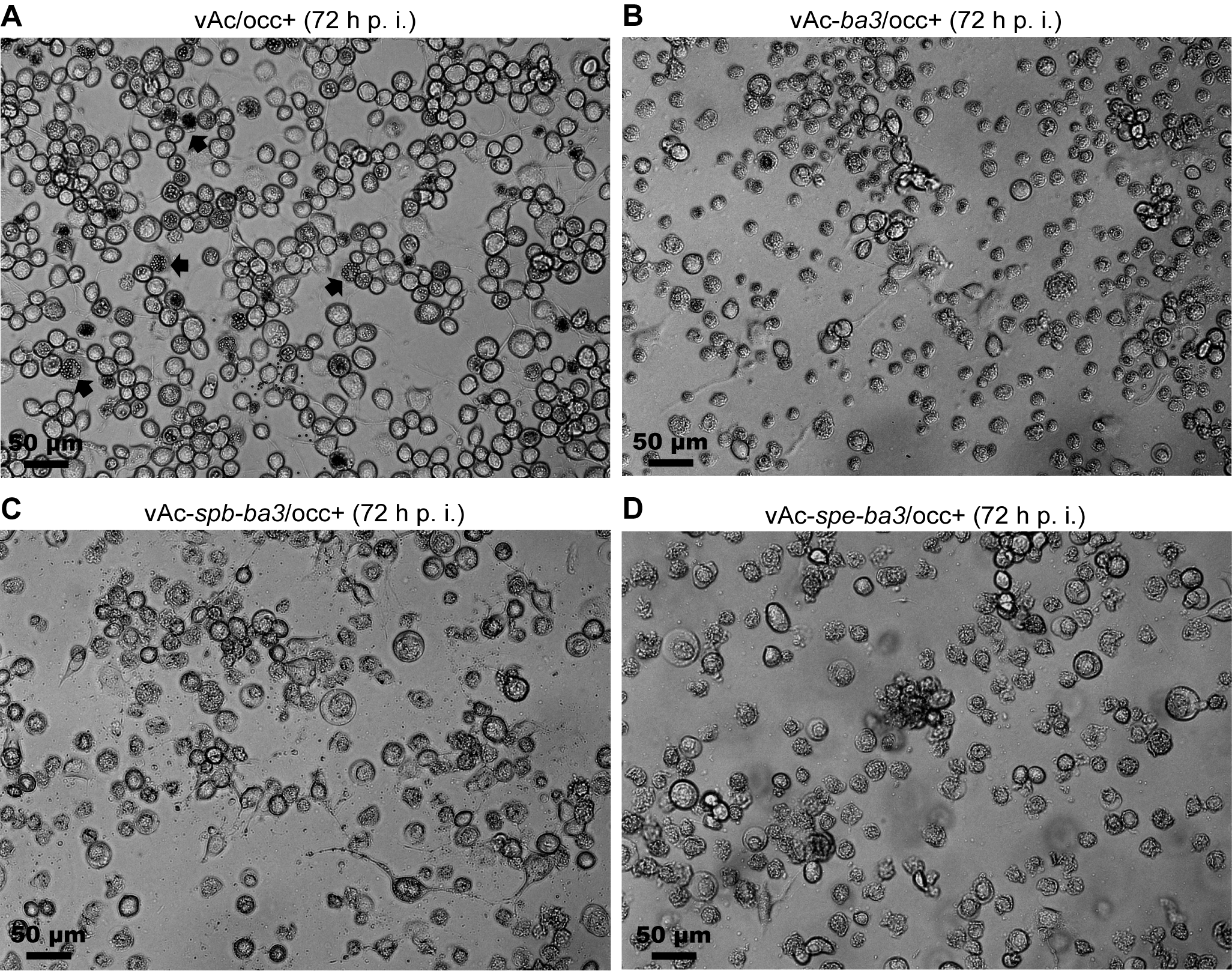

Supplement: Figure S4 — The Ba3 toxin causes early cell death by necrosis during recombinant baculovirus infections in Tn5B cells. Analysis of occlusion positive recombinant baculovirus infection by light microscopy of Tn5B monolayers infected with (A) vAc/occ+ (control virus), (B) the recombinant virus expressing the mature toxin Ba3, (vAc-ba3/occ+), (C) the recombinant virus expressing the mature toxin Ba3fused in frame with the bombyxin signal peptide (vAc-spb-ba3/occ+), or (D) with the egt signal peptide (vAc-spe-ba3/occ+). Even being occlusion positive, the recombinant viruses expressing the different variants of the toxin were not able to form polyhedra inside the nucleus as the control was (black arrows indicate polyhedra accumulation inside the cell nucleus). (TIF) [file pone.0084404.s005.tif]

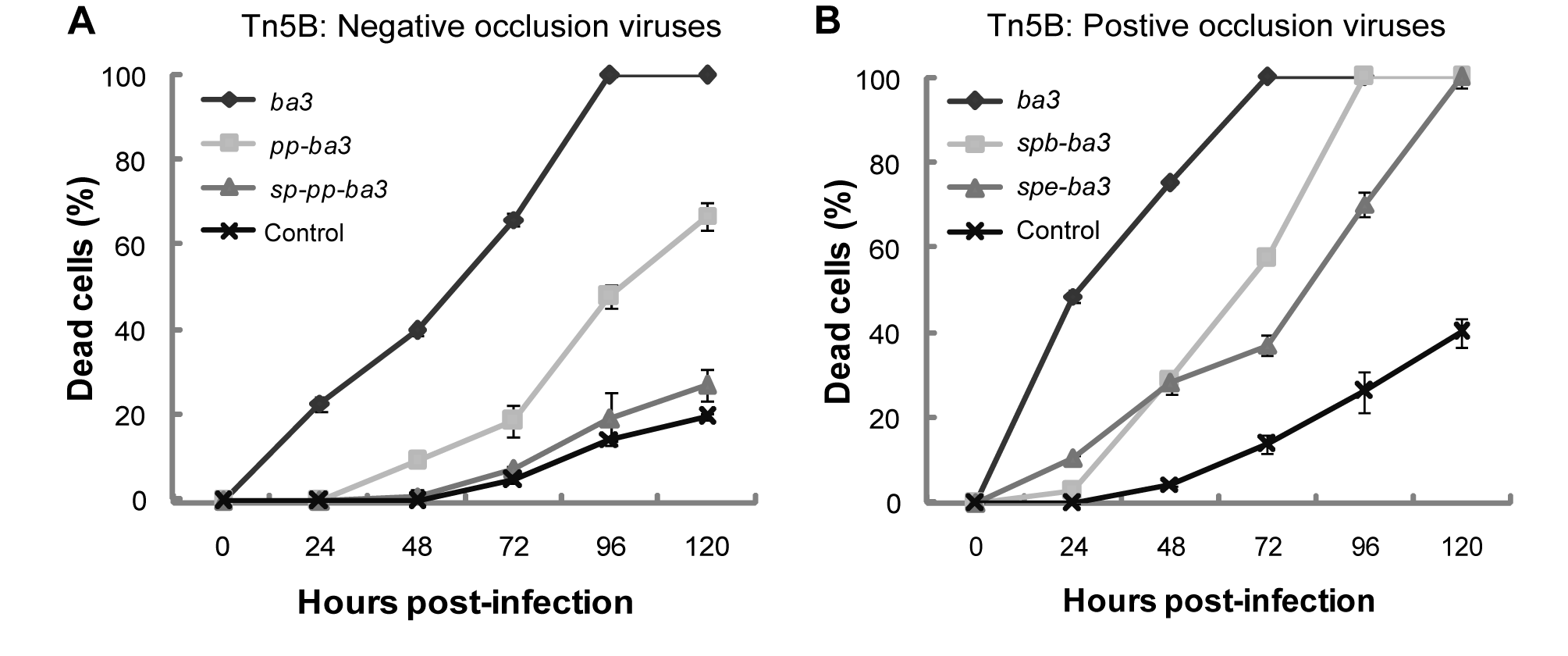

Supplement: Figure S5 — Cell viability quantification during infection of Tn5B cells with negative- and positive-occlusion recombinant baculovirus. (A) The recombinant virus containing the mature toxin gene (diamond –ba3), the propeptide (square - pp-ba3), the pre-propeptide (triangle - sp-pp-ba3) under the control of a very late promoter, or no toxin (x - Control) during infection of Tn5B cells at the indicated times post-infection (h p.i.). (B) The recombinant virus containing the mature toxin gene (diamond –ba3), the toxin gene fused in frame to a signal peptide derived from an insect gene (square - spb-ba3), or from a baculovirus gene (triangle - spe-ba3), all under the control of a late and very late promoter, or no toxin (x) during infection of Tn5B cells at the indicated times p.i. The results represent averages of at least three independent experiments observed at different times post-infection, and standard errors are indicated. The expression of the different version of the toxin under the control of two promoters (B) induced earlier cell death than the expression of the same toxins under the control of a very late promoter (A). (TIF) [file pone.0084404.s006.tif]
